# Supplementary material for: Molecular Design, Optimization, and Genomic Integration of Chimeric B Cell Receptors in Murine B Cells
Source: Front Immunol. 2019 Nov 14;10:2630. doi: 10.3389/fimmu.2019.02630 (PMC6868064; doi:10.3389/fimmu.2019.02630)
Supplement: Supplementary file 1 [file Data_Sheet_1.docx]

# Supplementary


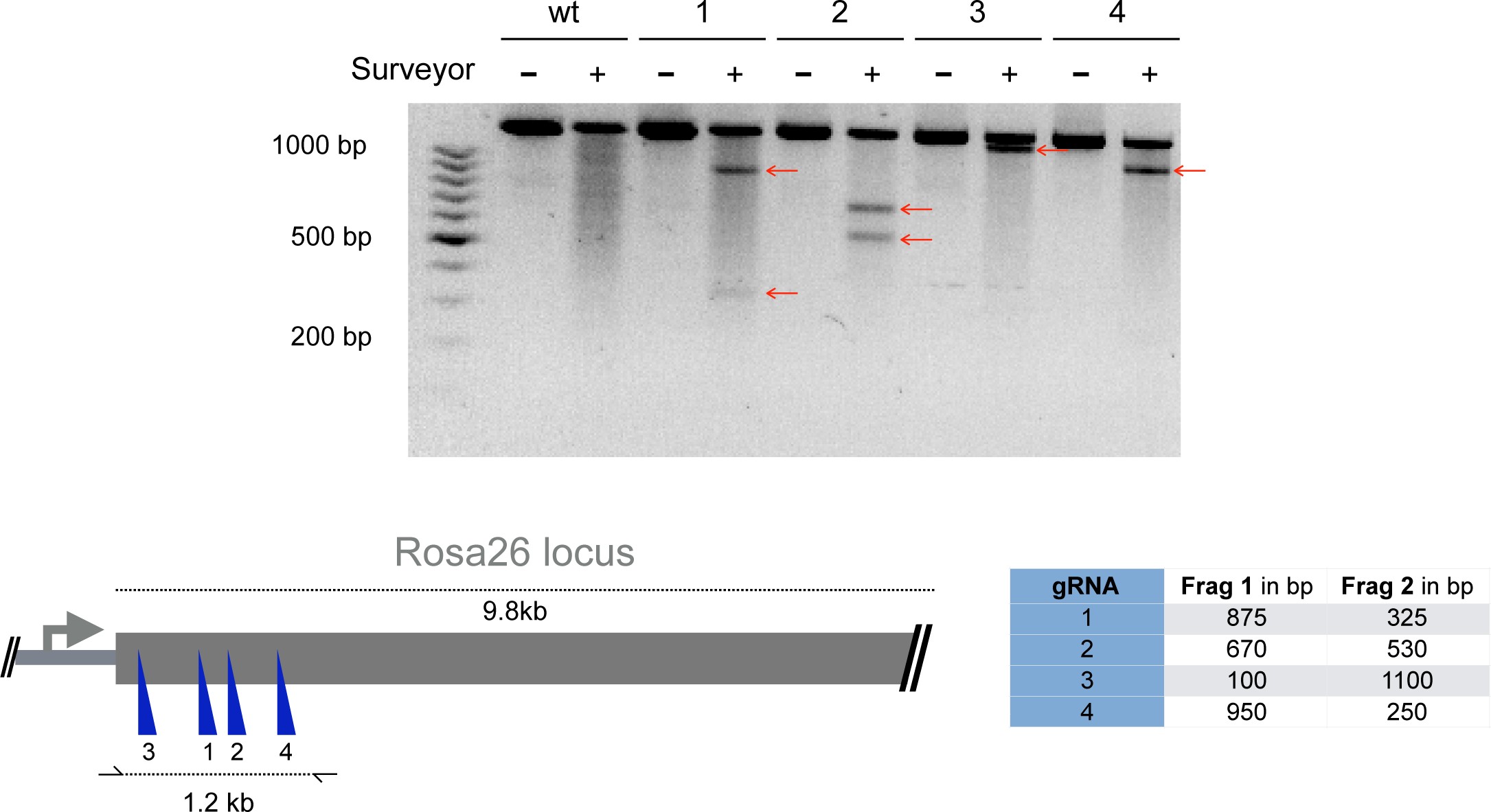


**Figure S1. Validation of CRISPR-Cas9 targeting in Rosa26 locus.** Surveyor assays following transfection of Cas9 protein complexed with gRNAs targeting the safe harbor Rosa26 locus. Non- transfected cells were used as negative control (wt). Table shows the approximate length of expected fragments after cleavage. Red arrows in agarose gel indicate cleavage products. gRNA1 was selected for further experiments.


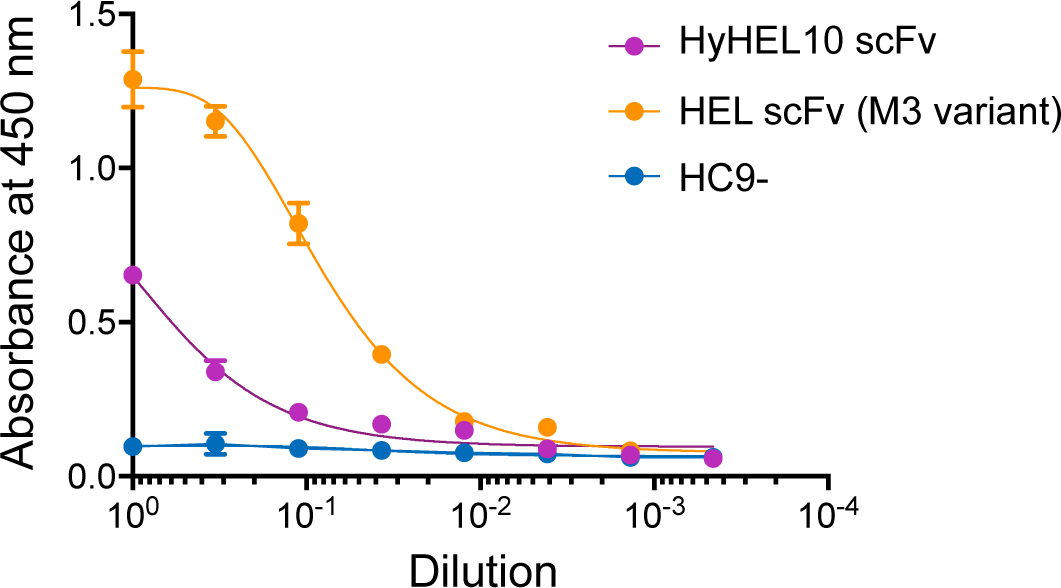


**Figure S2. Secretion test of HEL-binding scFv variants.** Cells were enriched for GFP (488 nm) expression via FACS. Graph shows ELISA results of scFv secretion levels (capture HEL antigen, detection anti-Flag) on enriched hybridoma culture supernatant for scFv variants, M3 variant of D1.3 (V_H_-V_L_ format) and HyHEL10 variant (V_L_-V_H_). Supernatant of HC9- cells was used as negative control. For each sample, three technical replicates were analysed and a four-parameter logistical curve was fitted to the data by nonlinear regression. Data are presented as the mean and error bars indicate standard deviation.


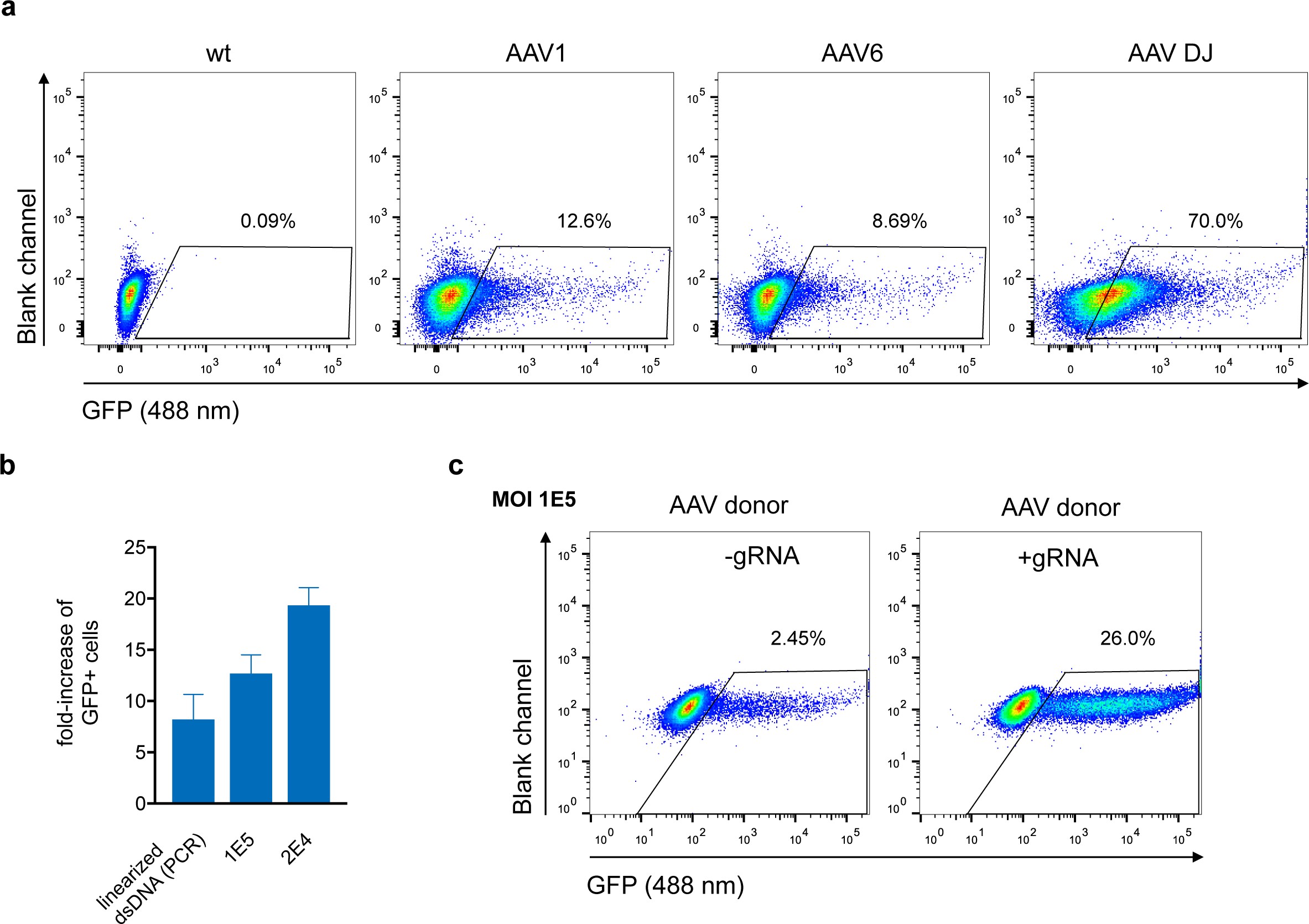


**Figure S3. Delivery of HDR donor DNA in the format of AAV. a**) Representative flow cytometry plots show the transduction efficiencies for serotype AAV-1, AAV-6 and AAV-DJ at a MOI of 10^5^ three days after transduction or mock treatment. **b**) HC9- cells were transfected with Cas9-RNP immediately followed by HDR repair template delivery via chimeric AAV serotype DJ encoding the GFP reporter gene. Cells transfected with Cas9-RNP and PCR-linearized dsDNA served as control. GFP expression was measured on day 9 after transfection. Cells transfected with Cas9-protein only indicate the episomal AAV background expression. Data are displayed as fold-increase of GFP^+^ cells receiving the Cas9-gRNA complex to cells transfected with Cas9-protein only representing the HDR based integration. All data are means ±s.d (n=3). **c**) Representative flow cytometry dot plots show GFP expression (488 nm) day for transduction with a MOI of 10^5^. Cells transfected only with Cas9-protein without gRNA and transduced with GFP expressing HDR donor packaged using scAAV-DJ were used as negative control to determine the background level of episomal GFP expression.


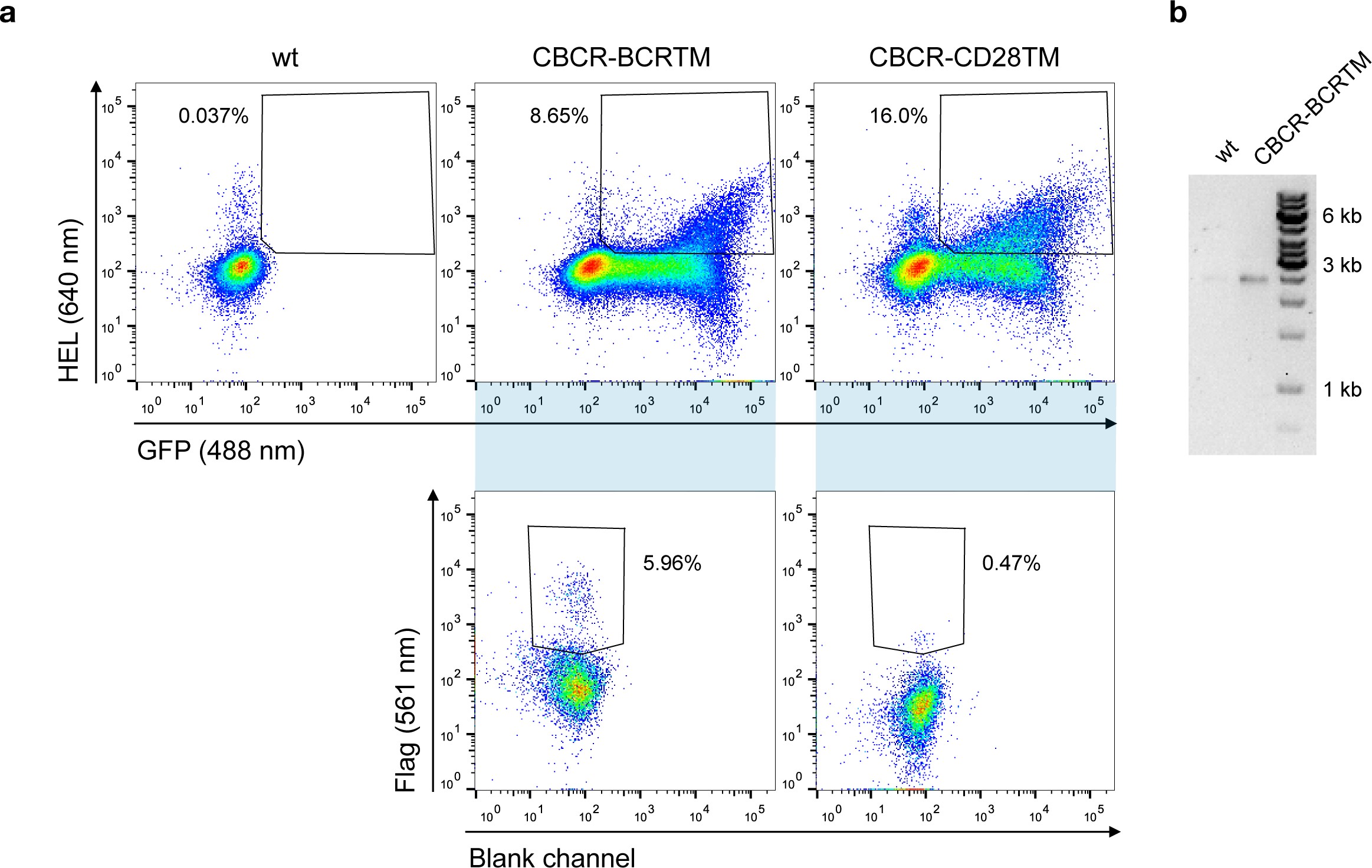


**Figure S4. Robust CBCR surface expression and antigen binding, but modest Flag detection in primary B cells. a**) Three days after transfection of splenic B cells with Cas9-RNP and repair templates encoding CBCR constructs with both TM domains and Flag detection tag within the extracellular spacer, GFP expressing cells (488 nm) were enriched by FACS. Flow cytometry dot plots show efficient enrichment of GFP+ cells and HEL antigen binding (upper row), but only modest detection of the Flag epitope (561 nm) within this population (lower row). **b**) Agarose gel shows PCR product from genomic DNA analysis (p7/15) that confirm the targeted integration of the GFP-T2A-CBCR-BCRTM into the Rosa26 locus (2442bp).

# Table S1: Primers used for DNA construct engineering and genomic analysis

| **Name** | **Binding region** | **Sequence (5**ʹ **to 3**ʹ**)** |
| --- | --- | --- |
| p1 (for) | GFP | ATGGAGATCGAGTGCCGCATCA |
| p2 (rev) | polyA | CACTGCGATTAAAGACATGCTCACCCG |
| p3 (for) | 5ʹ HA (Rosa26) | CAGATTTTCGGTTTTGTCGGG |
| p4 (rev) | 3ʹ HA (Rosa26) | GGATATGAAGTACTGGGCTCTT |
| p5 (for) | M3 scFV (V_H_) | ATCTGGGGAGACGGCAATAC |
| p6 (rev) | Rosa26 | AGTAATGGCTGCAGACTTAGCTTT |
| p7 (for) | Rosa26 | CCACCGCCCCACACTTATT |
| p8 (rev) | M3 scFv (V_H_) | ACCCAGTCAAGCTAAACCCG |
| p9 (for) | Rosa26 (5ʹ HA long) | CAGTTATGGCTCCTCTGTCCAC |
| p10(rev) | Rosa26 (3ʹ HA long) | CAGAGAGCCTCGGCTAGGT |
| p11(for) | Rosa26 (5ʹ HA AAV) | ATTATGTTAGCGGCCGCATTGGCTCGTGTTCGTGCA |
| p12(rev) | Rosa26 (3ʹ HA AAV) | ACTTCAGCGGCCGCCACCTTTTACAGATGTGTAC |
| p13 (for) | Rosa26 (Surveyor) | GTTCTCTGCTGCCTCCTG |
| p14 (rev) | Rosa26 (Surveyor) | CAAGCCAGTCCAAGAGAAAGCACTG |
| p15 (rev) | T2A | TTGAAGTCGCCGATCACG |

**Table S2: List of all gRNAs used in this study**

| **Name** | **Target region** | **Sequence (5**ʹ **to 3**ʹ**)** |
| --- | --- | --- |
| 1 | Rosa26 | CGATGGAAAATACTCCGAGG |
| 2 | Rosa26 | GTTACATACACCACAAATCG |
| 3 | Rosa26 | ACTCCAGTCTTTCTAGAAGA |
| 4 | Rosa26 | AGATACATCAGGTAATATTG |

**Table S3: Summary of cell lines used and generated in this study**

**Name Description**

Wen1.3 cells are derived from a mouse infected with LCMV. They express IgG2c

and are specific for LCMV GP-1 antigen.

Wen1.3

HC9- HC9- are a monoclonal population and contain a frameshift mutation in the CDRH3 resulting in dysfunctional antibody expression (PnP-HEL23.Fl^30^). It also includes constitutive Cas9 expression.

CBCR-CD79b Representative example (including the CD79b intracellular domain) for all cell lines containing a CBCR construct precisely integrated into the Rosa26 locus. CBCR constructs are constitutively expressed under the control of a CMV promoter.

CBCR expressing cells are derived from HC9-.

40LB 40LB are Balb/c 3T3 fibroblasts stably expressing CD40L and BAFF and used for

*in vitro* culture of primary murine B cells^48^.

**Table S4: Antibodies used for ELISA assays**

| **Target antigen** | **Conjugate** | **Host** | **Working concentration**  **(dilution from stock)** | **Cat. No, supplier** |
| --- | --- | --- | --- | --- |
| Myc tag (9E10) | HRP | mouse | 2 µg ml^-1^ (1:500) | MA1-81357, Thermo |
| Flag (FG4R) | HRP | mouse | 2 µg ml^-1^ (1:500) | MA1-91878-HRP, Thermo |

# Table S5: Flow cytometry labeling reagents with their working concentrations.

| **Target antigen** | **Conjugate** | **Working concentration**  **(dilution from stock)** | **Cat. No, supplier** |
| --- | --- | --- | --- |
| Flag (FG4R) | DyLight550 | 5 µg ml^-1^ (1:200) | MA1-91878-D550, Thermo |
| Strep tag II | Biotin | 2.5 µg ml^-1^ (1:200) | A01732, GenScript |
| Hen egg lysozyme (HEL) | AlexaFluor®647 | 1.6 µg ml^-1^ (1:200) | 62971-10G-F (Sigma  Aldrich) |
| Biotin | Brilliant Violet 421 | 0.2 µg ml^-1^ (1:500) | 405226, BioLegend |

**Protein and DNA sequences**

**Rosa26 locus** CCACCGCCCCACACTTATTGGCCGGTGCGCCGCCAATCAGCGGAGGCTGCCGGGGCCGCCTA AAGAAGAGGCTGTGCTTTGGGGCTCCGGCTCCTCAGAGAGCCTCGGCTAGGTAGGGGATCGGG ACTCTGGCGGGAGGGCGGCTTGGTGCGTTTGCGGGGATGGGCGGCCGCGGCAGGCCCTCCGA GCGTGGTGGAGCCGTTCTGTGAGACAGCCGGGTACGAGTCGTGACGCTGGAAGGGGCAAGCG GGTGGTGGGCAGGAATGCGGTCCGCCCTGCAGCAACCGGAGGGGGAGGGAGAAGGGAGCGG AAAAGTCTCCACCGGACGCGGCCATGGCTCGGGGGGGGGGGGGCAGCGGAGGAGCGCTTCC GGCCGACGTCTCGTCGCTGATTGGCTTCTTTTCCTCCCGCCGTGTGTGAAAACACAAATGGCGT GTTTTGGTTGGCGTAAGGCGCCTGTCAGTTAACGGCAGCCGGAGTGCGCAGCCGCCGGCAGCC TCGCTCTGCCCACTGGGTGGGGCGGGAGGTAGGTGGGGTGAGGCGAGCTGGACGTGCGGGC GCGGTCGGCCTCTGGCGGGGCGGGGGAGGGGAGGGAGGGTCAGCGAAAGTAGCTCGCGCGC GAGCGGCCGCCCACCCTCCCCTTCCTCTGGGGGAGTCGTTTTACCCGCCGCCGGCCGGGCCT CGTCGTCTGATTGGCTCTCGGGGCCCAGAAAACTGGCCCTTGCCATTGGCTCGTGTTCGTGCAA GTTGAGTCCATCCGCCGGCCAGCGGGGGCGGCGAGGAGGCGCTCCCAGGTTCCGGCCCTCCC CTCGGCCCCGCGCCGCAGAGTCTGGCCGCGCGCCCCTGCGCAACGTGGCAGGAAGCGCGCG CTGGGGGCGGGGACGGGCAGTAGGGCTGAGCGGCTGCGGGGCGGGTGCAAGCACGTTTCCG ACTTGAGTTGCCTCAAGAGGGGCGTGCTGAGCCAGACCTCCATCGCGCACTCCGGGGAGTGGA GGGAAGGAGCGAGGGCTCAGTTGGGCTGTTTTGGAGGCAGGAAGCACTTGCTCTCCCAAAGTC GCTCTGAGTTGTTATCAGTAAGGGAGCTGCAGTGGAGTAGGCGGGGAGAAGGCCGCACCCTTC TCCGGAGGGGGGAGGGGAGTGTTGCAATACCTTTCTGGGAGTTCTCTGCTGCCTCCTGGCTTCT GAGGACCGCCCTGGGCCTGGGAGAATCCCTTCCCCCTCTTCCCTCGTGATCTGCAACTCCAGTC TTTCTAGAAGATGGGCGGGAGTCTTCTGGGCAGGCTTAAAGGCTAACCTGGTGTGTGGGCGTTG TCCTGCAGGGGAATTGAACAGGTGTAAAATTGGAGGGACAAGACTTCCCACAGATTTTCGGTTTT GTCGGGAAGTTTTTTAATAGGGGCAAATAAGGAAAATGGGAGGATAGGTAGTCATCTGGGGTTTT ATGCAGCAAAACTACAGGTTATTATTGCTTGTGATCCGCCTCGGAGTATTTTCCATCGAGGTAGA TTAAAGACATGCTCACCCGAGTTTTATACTCTCCTGCTTGAGATCCTTACTACAGTATGAAATTAC AGTGTCGCGAGTTAGACTATGTAAGCAGAATTTTAATCATTTTTAAAGAGCCCAGTACTTCATATC CATTTCTCCCGCTCCTTCTGCAGCCTTATCAAAAGGTATTTTAGAACACTCATTTTAGCCCCATTT TCATTTATTATACTGGCTTATCCAACCCCTAGACAGAGCATTGGCATTTTCCCTTTCCTGATCTTA GAAGTCTGATGACTCATGAAACCAGACAGATTAGTTACATACACCACAAATCGAGGCTGTAGCTG GGGCCTCAACACTGCAGTTCTTTTATAACTCCTTAGTACACTTTTTGTTGATCCTTTGCCTTGATC CTTAATTTTCAGTGTCTATCACCTCTCCCGTCAGGTGGTGTTCCACATTTGGGCCTATTCTCAGTC CAGGGAGTTTTACAACAATAGATGTATTGAGAATCCAACCTAAAGCTTAACTTTCCACTCCCATGA ATGCCTCTCTCCTTTTTCTCCATTTATAAACTGAGCTATTAACCATTAATGGTTTCCAGGTGGATG TCTCCTCCCCCAATATTACCTGATGTATCTTACATATTGCCAGGCTGATATTTTAAGACATTAAAA GGTATATTTCATTATTGAGCCACATGGTATTGATTACTGCTTACTAAAATTTTGTCATTGTACACAT CTGTAAAAGGTGGTTCCTTTTGGAATGCAAAGTTCAGGTGTTTGTTGTCTTTCCTGACCTAAGGT CTTGTGAGCTTGTATTTTTTCTATTTAAGCAGTGCTTTCTCTTGGACTGGCTTGACTCATGGCATT CTACACGTTATTGCTGGTCTAAATGTGATTTTGCCAAGCTTCTTCAGGACCTATAATTTTGCTTGA CTTGTAGCCAAACACAAGTAAAATGATTAAGCAACAAATGTATTTGTGAAGCTTGGTTTTTAGGTT GTTGTGTTGTGTGTGCTTGTGCTCTATAATAATACTATCCAGGGGCTGGAGAGGTGGCTCGGAG TTCAAGAGCACAGACTGCTCTTCCAGAAGTCCTGAGTTCAATTCCCAGCAACCACATGGTGGCTC ACAACCATCTGTAATGGGATCTGATGCCCTCTTCTGGTGTGTCTGAAGACCACAAGTGTATTCAC ATTAAATAAATAAATCCTCCTTCTTCTTCTTTTTTTTTTTTTTAAAGAGAATACTGTCTCCAGTAGAA TTTACTGAAGTAATGAAATACTTTGTGTTTGTTCCAATATGGTAGCCAATAATCAAATTACTCTTTA AGCACTGGAAATGTTACCAAGGAACTAATTTTTATTTGAAGTGTAACTGTGGACAGAGGAGCCAT AACTGCAGACTTGTGGGATACAGAAGACCAATGCAGACTTTAATGTCTTTTCTCTTACACTAAGCA ATAAAGAAATAAAAATTGAACTTCTAGTATCCTATTTGTTTAAACTGCTAGCTTTACTTAACTTTTGT GCTTCATCTATACAAAGCTGAAAGCTAAGTCTGCAGCCATTACT

Grey: Homology arms Orange: PAM

Blue: CRISPR gRNA target site

Red: primer for genomic DNA analysis (p9 and p10)

**CMV promoter** ATAGTAATCAATTACGGGGTCATTAGTTCATAGCCCATATATGGAGTTCCGCGTTACATAACTTAC GGTAAATGGCCCGCCTGGCTGACCGCCCAACGACCCCCGCCCATTGACGTCAATAATGACGTAT GTTCCCATAGTAACGCCAATAGGGACTTTCCATTGACGTCAATGGGTGGAGTATTTACGGTAAAC TGCCCACTTGGCAGTACATCAAGTGTATCATATGCCAAGTCCGCCCCCTATTGACGTCAATGACG GTAAATGGCCCGCCTGGCATTATGCCCAGTACATGACCTTACGGGACTTTCCTACTTGGCAGTA CATCTACGTATTAGTCATCGCTATTACCATGGTGATGCGGTTTTGGCAGTACACCAATGGGCGTG GATAGCGGTTTGACTCACGGGGATTTCCAAGTCTCCACCCCATTGACGTCAATGGGAGTTTGTTT TGGCACCAAAATCAACGGGACTTTCCAAAATGTCGTAATAACCCCGCCCCGTTGACGCAAATGG GCGGTAGGCGTGTACGGTGGGAGGTCTATATAAGCAGAGGTCGTTTAGTGAACCGTCAGATC

**GFP** ATGGAGAGCGACGAGAGCGGCCTGCCCGCCATGGAGATCGAGTGCCGCATCACCGGCACCCT GAACGGCGTGGAGTTCGAGCTGGTGGGCGGCGGAGAGGGCACCCCCAAGCAGGGCCGCATGA CCAACAAGATGAAGAGCACCAAAGGCGCCCTGACCTTCAGCCCCTACCTGCTGAGCCACGTGAT GGGCTACGGCTTCTACCACTTCGGCACCTACCCCAGCGGCTACGAGAACCCCTTCCTGCACGC CATCAACAACGGCGGCTACACCAACACCCGCATCGAGAAGTACGAGGACGGCGGCGTGCTGCA CGTGAGCTTCAGCTACCGCTACGAGGCCGGCCGCGTGATCGGCGACTTCAAGGTGGTGGGCAC CGGCTTCCCCGAGGACAGCGTGATCTTCACCGACAAGATCATCCGCAGCAACGCCACCGTGGA GCACCTGCACCCCATGGGCGATAACGTGCTGGTGGGCAGCTTCGCCCGCACCTTCAGCCTGCG CGACGGCGGCTACTACAGCTTCGTGGTGGACAGCCACATGCACTTCAAGAGCGCCATCCACCC CAGCATCCTGCAGAACGGGGGCCCCATGTTCGCCTTCCGCCGCGTGGAGGAGCTGCACAGCAA CACCGAGCTGGGCATCGTGGAGTACCAGCACGCCTTCAAGACCCCCATCGCCTTCGCCAGATC CCGCGCTCAGTCGTCCAATTCTGCCGTGGACGGCACCGCCGGACCCGGCTCCACCGGATCTCG C

MESDESGLPAMEIECRITGTLNGVEFELVGGGEGTPKQGRMTNKMKSTKGALTFSPYLLSHVMGYG FYHFGTYPSGYENPFLHAINNGGYTNTRIEKYEDGGVLHVSFSYRYEAGRVIGDFKVVGTGFPEDSVI FTDKIIRSNATVEHLHPMGDNVLVGSFARTFSLRDGGYYSFVVDSHMHFKSAIHPSILQNGGPMFAFR RVEELHSNTELGIVEYQHAFKTPIAFARSRAQSSNSAVDGTAGPGSTGSR

# T2A

GGCAGTGGAGAGGGCAGAGGAAGTCTGCTAACATGCGGTGACGTCGAGGAGAATCCTGGCCCA GSGEGRGSLLTCGDVEENPGP

# Signal peptide

ATGGTTTTCACACCTCAGATACTTGGACTTATGCTTTTTTGGATTTCAGCCTCCAGAGGT MVFTPQILGLMLFWISASRG

**HEL scFv (HyHEL10 derived)** GATATTGTGCTAACTCAGTCTCCAGCCACCCTGTCTGTGACTCCAGGAAATAGCGTCAGTCTTTC CTGCAGGGCCAGCCAAAGTATTGGCAACAACCTACACTGGTATCAACAAAAATCACATGAGTCTC CAAGGCTTCTCATCAAGTATGCTTCCCAGTCCATCTCTGGCATCCCCTCCAGGTTCAGTGGCAGT GGATCAGGGACAGATTTCACTCTCAGTATCAACAGTGTGGAGACTGAAGATTTTGGAATGTATTT CTGTCAACAGAGTAACAGCTGGCCGTACACGTTCGGAGGGGGGACCAAGCTGGAAATAAAAGG TGGTGGCGGTTCTGGCGGCGGTGGCTCCGGTGGTGGCGGCAGCGGTGGTGGCGGTAGCGAC GTGCAGCTTCAGGAGTCAGGACCTAGCCTCGTGAAACCTTCTCAGACTCTGTCCCTCACCTGTT CTGTCACTGGCGACTCCATCACCAGTGATTATTGGAGCTGGATCAGGAAATTCCCAGGGAATAG ACTTGAGTACATGGGGTACGTAAGCTACAGTGGTAGCACGTACTACAATCCATCTCTCAAAAGTC GAATCTCCATCACTCGAGACACATCCAAGAATCAGTACTATCTGGACCTGAATTCTGTGACTACT GAGGACACAGCCACATATTACTGTGCAAACTGGGACGGTGATTACTGGGGCCAAGGGACTCTG GTCACTGTCTCTGCA

DIVLTQSPATLSVTPGNSVSLSCRASQSIGNNLHWYQQKSHESPRLLIKYASQSISGIPSRFSGSGSG TDFTLSINSVETEDFGMYFCQQSNSWPYTFGGGTKLEIKGGGGSGGGGSGGGGSGGGGSDVQLQ

ESGPSLVKPSQTLSLTCSVTGDSITSDYWSWIRKFPGNRLEYMGYVSYSGSTYYNPSLKSRISITRDT SKNQYYLDLNSVTTEDTATYYCANWDGDYWGQGTLVTVSA

Blue: V_L_

Grey: GGGGS linker Orange: V_H_

**HEL scFv (D1.3 variant M3 derived)** CAGGTACAATTGCAAGAATCAGGACCAGGATTGGTTGCTCCCTCACAGTCCCTGTCCATCACAT GCACCGTGAGCGGGTTTAGCTTGACTGGGTATGGTGTCAATTGGGTTAGACAACTGCCTGGCAA GGGACTCGAGTGGCTCGGCATGATCTGGGGAGACGGCAATACAGCATATAACTCAGCATTGAAA AGTCGGTTGAGCATATCTAAGGACAACAGCAAATCCCAAGTATTTCTTAAGATGGATTCTTTGCA CACTGACGACACTGCACGCTACTACTGCGCTCGGGAACGGGATTACAGATTGGATTATTGGGGA CAGGGAACTACTGTAACCGTTAGCTCTGGTGGCGGAGGGTCAGGAGGTGGTGGTTCAGGTGGT GGCGGGAGTGACATCAAATTGACCCAATCTCCAGCAAGTTTGAGCGCTTCAGTAGGAGAAACAG TTACAATCACCTGTAGAGCCTCCGGGAATACTCACAATTACCTTGCCTGGTATCAACAGAAACAA GGAAAGTCCCCACAGCTCTTGGTATATTACACTACAACACTTGCTGACGGAGTTCCCTCAAGGTT TAGCGGTTCCGGGAGCGGAACACAGTATTCACTCAAGATCAACTCACTGCAACCAGAAGATTTT GGAAGCTACTACTGTCAACACTTCTGGTCTACCCCTCGCTCCTTTGGGGGTGGCACCAAATTGG AAATCAAACGG

QVQLQESGPGLVAPSQSLSITCTVSGFSLTGYGVNWVRQLPGKGLEWLGMIWGDGNTAYNSALKS RLSISKDNSKSQVFLKMDSLHTDDTARYYCARERDYRLDYWGQGTTVTVSSGGGGSGGGGSGGG GSDIKLTQSPASLSASVGETVTITCRASGNTHNYLAWYQQKQGKSPQLLVYYTTTLADGVPSRFSGS GSGTQYSLKINSLQPEDFGSYYCQHFWSTPRSFGGGTKLEIKR

# Extracellular spacer

ECL23 GGCGCCGGATCTGGAGGAGACTACAAGGACGATGACGACAACGGCGGTTCGGGTGGATCGGG GGGAGGT

GAGSGGDYKDDDDNGGSGGSGGG

## ECL26

GGCGCCGGATCTGGAGGAGACTACAAGGACGATGACGACAACGGTGGTAGTGGCGGTTCGGG TGGATCGGGGGGAGGT

GAGSGGDYKDDDDNGGSGGSGGSGGG

## ECL32

GGCGCCGGATCTGGAGGAGACTACAAGGACGATGACGACAACGGCGGATCGGGGGGGTCTGG TGGTAGTGGCGGTTCGGGTGGATCGGGGGGAGGT

GAGSGGDYKDDDDNGGSGGSGGSGGSGGSGGG

## Linker with Strep tag II (1x)

GGCGCCGGATCTGGAGGAAACTGGTCACACCCACAGTTCGAGAAGGGTGGTAGTGGCGGTTCG GGTGGATCGGGGGGAGGT

GAGSGGNWSHPQFEKGGSGGSGGSGGG

## Linker with Strep tag II (3x)

GGCGCCGGATCTGGAGGAAACTGGTCACACCCACAGTTCGAGAAGGGTGGTAGTGGCGGTTCG GGTGGAAGTGGAGGTTCGAACTGGTCGCACCCACAGTTCGAGAAGGGGGGGAGCGGGGGCTC AGGCGGTAGTGGGGGCTCTAACTGGTCCCACCCACAGTTCGAGAAGGGTGGATCGGGGGGAG GT

GAGSGGNWSHPQFEKGGSGGSGGSGGSNWSHPQFEKGGSGGSGGSGGSNWSHPQFEKGGSG GG

Orange: Flag tag Red: Strep tag II

**BCR (IgG2c)-TM** CTAGACCTGGATGATATCTGTGCTGAGGCCAAGGATGGGGAGCTGGACGGGCTCTGGACGACC ATCACCATCTTCATCAGCCTCTTCCTGCTCAGCGTGTGCTACAGCGCCTCTGTCACACTCTTCAA GGTA

LDLDDICAEAKDGELDGLWTTITIFISLFLLSVCYSASVTLFKV

**CD28-TM** TTCTGGGCCCTGGTGGTGGTGGCCGGCGTGCTGTTCTGTTACGGCCTGCTGGTCACAGTGGCC CTGTGCGTGATCTGGACC

FWALVVVAGVLFCYGLLVTVALCVIWT

**BCR-IC** AAGTGGATCTTCTCCTCAGTGGTGGAGCTGAAGCAGAAGATCTCCCCTGACTACAGAAACATGA TTGGGCAGGGAGCC

KWIFSSVVELKQKISPDYRNMIGQGA

**CD79α-IC** AGGAAGAGGTGGCAGAACGAAAAATTTGGGGTAGACATGCCAGATGACTACGAGGATGAGAATC TGTATGAAGGTCTCAATCTTGACGATTGCTCAATGTACGAAGACATATCCAGAGGGCTCCAGGG CACTTACCAGGATGTCGGCAATCTGCATATAGGAGATGCCCAGCTTGAGAAGCCC

RKRWQNEKFGVDMPDDYEDENLYEGLNLDDCSMYEDISRGLQGTYQDVGNLHIGDAQLEKP

Yellow: ITAM

**CD79β-IC** GACAAAGACGATGGTAAAGCCGGAATGGAAGAAGATCATACCTATGAAGGTCTTAATATCGACCA AACAGCAACCTACGAGGACATCGTAACACTTCGCACAGGCGAAGTGAAATGGAGCGTTGGAGAG CACCCCGGTCAGGAG

DKDDGKAGMEEDHTYEGLNIDQTATYEDIVTLRTGEVKWSVGEHPGQE
